# Sequential Determination of Total Arsenic and Cadmium in Concentrated Cadmium Sulphate Solutions by Flow-Through Stripping Chronopotentiometry after Online Cation Exchanger Separation

**DOI:** 10.1155/2012/814983

**Published:** 2012-01-12

**Authors:** Frantisek Cacho, Lukas Lauko, Alena Manova, Jan Dzurov, Ernest Beinrohr

**Affiliations:** ^1^Institute of Analytical Chemistry, Slovak University of Technology, Radlinského 9, 812 37 Bratislava, Slovakia; ^2^Istran Ltd., Zitna 42, 831 06 Bratislava, Slovakia; ^3^Department of Chemistry, Faculty of Natural Sciences, University of SS. Cyril and Methodius in Trnava, Jozefa Herdu 2, 917 01 Trnava, Slovakia

## Abstract

Flow-through stripping chronopotentiometry with a gold wire electrode was used for the determination of total arsenic and cadmium in cadmium sulphate solutions for cadmium production. The analysis is based on the online separation of arsenic as arsenate anion from cadmium cations by means of a cation exchanger. On measuring arsenate in the effluent, the trapped cadmium is eluted by sodium chloride solution and determined in a small segment of the effluent by making use of the same electrode. The elaborated protocol enables a full automatic measurement of both species in the same sample solution. The accuracy of the results was confirmed by atomic absorption spectrometry. The LOD and LOQ for Arsenic were found to be 0.9 *μ*g dm^−3^ and 2.7 *μ*g dm^−3^, respectively. A linear response range was observed in the concentration range of 1 to 300 *μ*g dm^−3^ for sample volumes of 4 mL. The repeatability and reproducibility were found to be 2.9% and 5.2%, respectively. The linear response range for cadmium was found to be 0.5 to 60 g/L. The method was tested on samples from a cadmium production plant.

## 1. Introduction

The extraction of cadmium from the technological liquids coming from zinc production is mostly done by chemical reduction of cadmium from acidic solutions by means of powdered zinc. The reduction and flocculation of cadmium significantly depends on pH value and cadmium concentration. Arsenic contents in these liquids may enhance the risks of the formation of toxic arsenic hydride (arsine) which must absolutely be eliminated for safety reasons. Hence, a rapid information about cadmium and arsenic concentrations is of immense importance, the former for technological and the latter for safety reasons.

There are numerous analytical methods for the determination of arsenic [1, 2] and cadmium [3] in various sample matrices. Unfortunately, only few of them are rugged enough to be used in field operation, directly in the production area. Moreover, measurement of low arsenic contents in the presence of large excess of interfering cadmium and/or similar metal ions such as Pb, Sb, Bi, and Cu cannot be performed by conventional methods such as atomic absorption spectroscopy or spectrophotometry.

Electrochemical methods offer several advantages over spectroscopic ones; namely, much simpler instrumentation, fast and sensitive response, broad concentration range significantly lower costs, and electrochemical methods are amenable to easy automation. Matrix interferences and lower selectivity, however, may be a serious problem. Arsenic and cadmium can easily be measured by voltammetric and chronopotentiometric methods making the methods suitable for the task outlined above. However, a direct measurement of arsenic in the presence of large excess of cadmium is not feasible owing to the interfering effect of the latter. Separation of arsenic from cadmium is therefore a prerequisite for an interference-free determination of arsenic.

Arsenic can be separated from cadmium and similar metal ions by making use of the volatility of arsenic halides, especially AsCl_3_ [4, 5]. Here, the volatile arsenic chloride is distilled from the strongly acidified solution into a trapping solution and is subsequently determined, whereas the interfering metal ions remain in the original solution. The distillation procedure is time consuming and the procedure is laborious making the method less suitable for routine use. Extraction of arsenic chloride from concentrated hydrochloric acid media into a suitable organic solvent has also been used for the separation of arsenic [6] as well as supercritical extraction followed by voltammetric determination of arsenic [7]. Again, the laborious procedure hinders its application in field analysis. Arsenic in these solutions is present prevailing as arsenite and arsenate, that is, as an anion, which enables its separation from interfering metal ions-cations by making use of ion exchangers. Both cation and anion exchangers can be used. Cadmium is retained in the former, arsenic in the latter. However, in the presence of a large excess of a cadmium salt, cadmium sulphate in this case, the excess anion competes with arsenate/arsenite anions hindering their separation from cadmium. Hence, a cation exchanger with sufficient capacity and in H^+^ or Na^+^ cycle can only be used: arsenite/arsenate species pass the ionex whereas Cd^2−^ ions remain in it being replaced by H^+^ or Na^+^ ions. Arsenic then can be measured in the effluent virtually containing no cadmium. On eluting cadmium from the ionex by a concentrated HCl or NaCl solution the cadmium content can also be determined. At the same time the ionex will be regenerated and made ready for the next run.

## 2. Experimental

### 2.1. Apparatus

**Figure 1:**
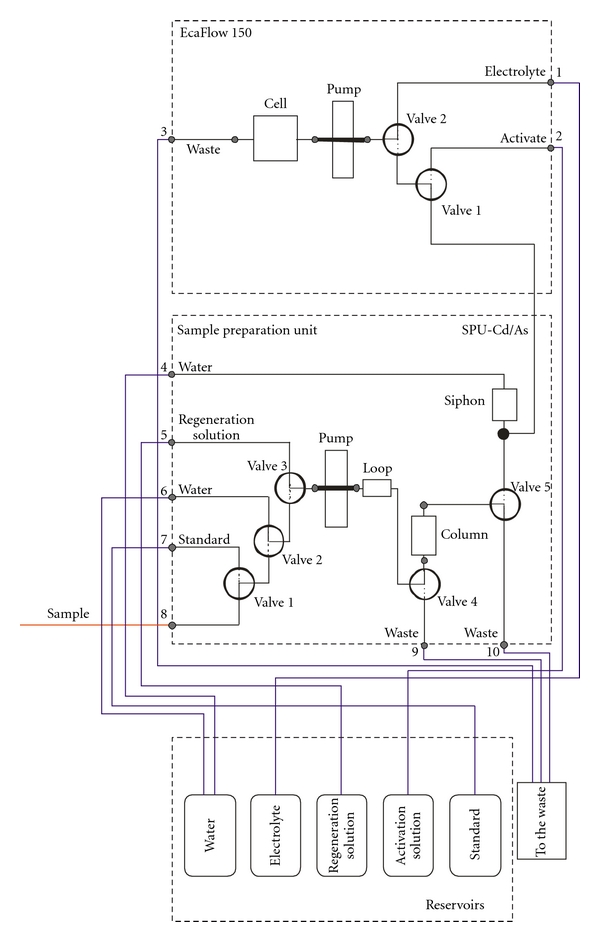
Flow chart diagram of the Cd/As measuring system. See explanation in the text.

The Cd/As-measuring system comprises a commercial flow-through electrochemical analyser EcaFlow 150 (Istran, Ltd., Bratislava, Slovakia), the online separation unit SPU, and the control PC. The flow chart diagram of the system is depicted in Figure 1. 

The electrochemical analyser EcaFlow 150 was equipped with two solenoid inert valves, a peristaltic pump and a microprocessor-controlled potentiostat/galvanostat. The peristaltic pump was switched to one of the three input solutions (carrier electrolyte or activation solution or input from the SPU). A compact flow-through electrochemical cell of type 353c with Pt auxiliary, Ag/AgCl reference, and E-T/Au-working electrode was used (Istran, Ltd., Bratislava, Slovakia). The working electrode was a gold wire of 0.5 mm and 4 mm in diameter and length, respectively. The electrode was positioned perpendicularly to the flow. The operation parameters are listed in Table 1. 

The online separation unit SPU comprises three solenoid valves 075T3MP (Bio-Chem Fluidics, Cambridge, UK), to switch to the sample, calibration solution, rinsing water, and column regeneration solution. One of these solutions was delivered by the peristaltic pump ISM 144 (ISMATEC SA, Glattbrugg, Switzerland), either to the waste or pumped through the separation column. From the separation column the solution could be directed either to the waste or to the overflow (siphon) where the solution could be taken up by the electrochemical analyser. The loop positioned between the pump and solenoid valve No. 4 was a coil of PTFE tube of 1 mm and 500 mm in diameter and length, respectively. Its role was to mix online the sample or standard with water to achieve suitable dilution degree. The siphon was made of a PTFE tube of 1.5 mm and 1000 mm in diameter and length, respectively. The separation column of 5 mm and 30 mm in inner diameter and length, respectively, was packed with a strong acidic cation exchanger Dowex 50 W in Na^+^ cycle with particle sizes of 75–150 *μ*m (Fluka AG, Buchs SG). 

Both units were controlled independently by microprocessors and the whole system was controlled by a PC by making use of a laboratory made software written in C+. The PC communicated with the electrochemical analyser and the SPU unit via the serial and parallel ports, respectively. 

The system was tested with samples from a cadmium production plant (Portovesme s. r. l., Portoscuso, Italy) with arsenic and cadmium contents of 0 to 500 *μ*g/L and of 0.5 to 50 g/L, respectively. The pH of the samples varied in the range of about 5 to 8. 

The accuracy of the results for As and Cd was checked by hydride generation AAS and flame atomization AAS, respectively, on the atomic absorption spectrometer Perkin Elmer 5000 (Germany). The experimental parameters used were those recommended by the manufacturer. 

### 2.2. Reagents and Solutions

Analytical grade reagents were used in all experiments. Deionised and degassed water was used for the preparation of all solutions:

- carrier electrolyte: 0.1 mol dm^−3^ HCl with 0.02% (V/V) Triton X-100 (Sigma Aldridge),
- column regeneration solution: 3.0 mol dm^−3^ NaCl,
- electrode activation solution: 0.01 mol dm^−3^ HAuCl_4_ in 1.0 mol dm^−3^ HCl,
- calibration solutions: Arsenic (10–200 ug dm^−3^) in cadmium sulphate solutions(1–50 g dm^−3^ Cd).

## 3. Results and Discussion

### 3.1. Arsenic Measurement

The measurement of arsenic in aquatic samples by stripping chronopotentiometry is well documented [8]. The electrochemical deposition of As(III) species is easy on gold or gold-coated electrodes and that from neutral and acidic solutions, preferably containing chloride ions. The use of silver electrode, however, in nitric acid media was also demonstrated [9]. Unlike the As(III) species, As(V) ones cannot be deposited as easily owing to the irreversibility of the As(V)/As(0) couple. Usually a time-consuming chemical prereduction step should be employed to convert As(V) to the electrochemically more active As(III). Obviously, for online sample treatment such a procedure is virtually useless. Procedures making use of heated electrodes [10], addition of complex forming reagents [11], and concentrated hydrochloric acid [12] are either inconvenient or require aggressive or toxic reagents. 

A simpler solution is to form a fresh gold layer on a carbon or gold electrode by electrolytic deposition of gold from and acidic gold solution [13]. Such an electrode surface facilitates the electrodeposition both of As(III) and As(VI) and hence enables the determination of total arsenic content without any chemical prereduction step even from less acidic solutions. Unfortunately, the lifetime of such a modified electrode is limited to 30–50 measurements; thereafter the sensitivity starts to drop. Nevertheless, a repeated coating restores the original sensitivity again; so the electrode can be used for several hundreds of measurement cycles. 

There is a difference in the sensitivities of the measurement of As(III) and As(V), the former being 30–50% higher than the latter. For total arsenic measurements all arsenic species are therefore converted to As(V) by addition of a permanganate solution to the sample. The oxidation is immediate and the excess of permanganate does not interfere. 

The measurement of arsenic by means of flow-through stripping chronopotentiometry was optimised and described earlier [14]. The determination is simple and sensitive enough but some heavy metal ions such as lead, copper, bismuth, and cadmium interfere. A direct measurement of As cadmium solutions is therefore not possible.

### 3.2. Cadmium Measurement

The determination of cadmium on the same electrode as that for arsenic is possible in the presence of arsenic only if the deposition potential is set to a value where arsenic is not yet deposited. Unfortunately from acidic solutions and on the gold electrode surface both species are deposited and their stripping peaks overlap slightly. Nevertheless, owing to the significantly lower content of arsenic, its potential interference would be negligible. 

### 3.3. Separation of Arsenic from Cadmium

Arsenic and cadmium species can easily be separated by means of cation exchangers. Being cadmium a cation it is trapped by a cation exchanger in H^+^ or Na^+^ cycle, whereas arsenic species being anions or neutral species (depending on pH and oxidation state of arsenic) pass through the ionex. Hence, arsenic can be measured in the effluent from the cation exchanger column. In the next step, the trapped cadmium can be eluted by a concentrated NaCl solution and measured. On washing the column with water it is ready for the next measurement. 

The dimensions of the column and the flow rate should be set to ensure a complete separation of cadmium ions. On the other side, the column should be small enough to minimise the rinsing time and the consumption of reagent solutions. For the applied flow rate (about 3 mL/min), columns of 5 mm and 25 mm in diameter and length, respectively, were found to fulfill the above requirements. 

### 3.4. Timing

Owing to the extremely different concentrations of arsenic and cadmium the SPU unit should ensure minimum dispersion for the arsenic zone and maximum dispersion for the eluted cadmium zone. This can be achieved by suitable timing of the valves directing the flows either to the column or to the waste or to the siphon. Each step was therefore optimised and built into the measurement algorithm. The detailed timing and measurement sequence is listed in Table 2. By making use of this protocol, an analysis is completed within 10–12 min. After completing 30 analyses, the electrode was automatically activated by depositing a fresh layer of gold from the activation solution at 400 mV for 5 min. 

### 3.5. Analytical Figures of Merit

**Figure 2:**
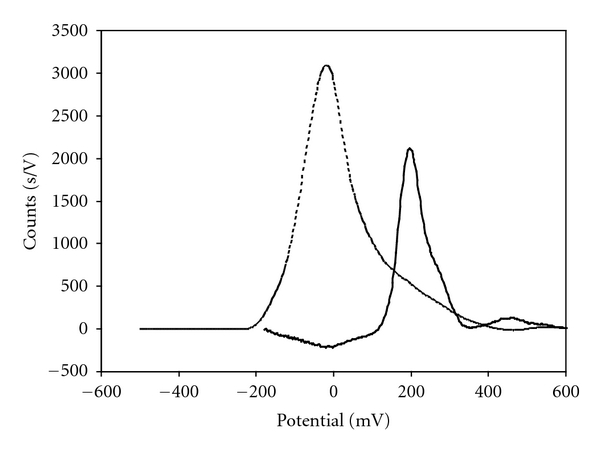
Background corrected chronopotentiometric signals obtained successively for As (full line) and Cd (broken line) by analysing a cadmium sulphate solution containing 100 *μ*g/L and 20 g/L of As and Cd, respectively. See experimental parameters in Section 2.

Typical signals for arsenic and cadmium are listed in Figure 2. The figures of merit are listed in Table 3. The limits of detection and determination for arsenic are higher than in solutions not containing large excess of cadmium ions but are still valuable. Namely, the threshold limit for arsenic contents in the treated technological solutions is 200 *μ*g/L; so concentrations near and below this value can reliably be measured. In spite of the complex sample pretreatment good repeatability and reproducibility were experienced. 

For cadmium determination the procedure had to ensure an efficient and reproducible dilution to get a final solution with reasonably low cadmium concentrations, preferably in the middle mg/L region. Fortunately the linear response range for cadmium is significantly broader than that for arsenic, comprising a concentration range of 0.01 to about 5 mg/L. However, for higher cadmium concentrations the cadmium peaks split, presumably due to multilayer deposition on the gold electrode surface. The amount of cadmium taken for the analysis is controlled by dispersion of the cadmium concentration in the eluted sample from the column and on the volume of this solution diverted to the sample reservoir. The found optimal timing ensured sufficient dilution degree and acceptable reproducibility. By making use of these parameters, a linear response range was observed for cadmium contents up to 60 g/L which met the requirements of the technology.

### 3.6. Real Samples

Real samples of concentrated cadmium solutions were obtained from a cadmium producing plant, where cadmium is gained as a by-product in the production of zinc. The samples were filtered and analysed directly by the elaborated procedure. The accuracy of the results was checked by hydride generation AAS and flame AAS for arsenic and cadmium, respectively. The determination of arsenic contents below 50–100 *μ*g/L (depending on the cadmium concentration) by HGAAS was not possible owing to the interfering effect of cadmium ions being in a huge excess. Some typical data obtained by the chronopotentiometric and control methods are listed in Table 4. Both methods delivered similar data for all samples, except for those with arsenic concentrations above 300 *μ*g/L, where the value obtained by FAAS was significantly higher (e.g., samples D and H in Table 4). This is due to the fact that the linear response range for arsenic comprises the range of 300 *μ*g/L only. Nevertheless, after a proper dilution of such samples with water, accurate results were obtained also for arsenic. The accuracy of arsenic measurement in samples with arsenic concentrations below 80 *μ*g/L was proved by spiking the samples with arsenic which resulted 85–115% recoveries.

## 4. Conclusions 

The elaborated method and flow system enables a reliable measurement of trace arsenic in concentrated cadmium solutions and at the same time the determination of the cadmium content as well. The completely automated procedure simplifies the analysis and enhances the precision of the data. The sample preparation is simple consisting of filtration of the sample and addition of a small volume of permanganate solution. 

The method could also be utilised for the determination of arsenic in the presence of any cationic species such as zinc, lead, and copper solutions.

## Figures and Tables

**Table 1:** Operation parameters of the electrochemical analyser.

Parameter	Value	
	Arsenic measurement	Cadmium measurement
Deposition potential, mV	−1200	
Deposition current, mA		−5
Quiescence potential 1, mV	−200	−1000
Quiescence time 1, s	10	5
Quiescence potential 2, mV	−100	−1000
Quiescence time 2, s	10	5
Terminal potential, mV	600	600
Stripping current, *μ*A	20	200
Stand by potential, mV	−500	−500
Sample volume, mL	5	2

**Table 2:** Timetable of the separation unit and the measurement sequence.

Step	Action	Note
1	Filling the sampling loop with sample or standard, the excess is directed to the waste though the valve No. 4. Duration 90 s	
2	Pumping the sample/standard from the sampling loop through the column to the reservoir. Duration 90 s	
3	A ready signal is sent to start the uptake of the sample by the analytical unit. The content of the reservoir is taken to the analytical unit and arsenic is measured	Arsenic measurement
4	The column is rinsed with the regeneration solution which elutes cadmium. The eluted solution is directed to the waste. Duration 110 s	
5	A zone of the eluted solution is diverted to the reservoir through the valve No. 5 (duration 20 s)	
6	A ready signal is sent to start the uptake by the analytical unit. The content of the reservoir is taken to the analytical unit and cadmium is measured	Cadmium measurement
7	In the meantime the column is rinsed with the regeneration solution and the solution is lead to the waste through the valve No. 5. Duration 150 s	
8	The column is rinsed with water. Duration 180 s	
9	The SPU is set to standby and is waiting for the next start	

**Table 3:** Analytical figures of merit.

Parameter	Value	
	Arsenic	Cadmium
Limit of detection	0.9 *μ*g/L	—
Limit of determination	2.7 *μ*g/L	—
Linear range	3–300 *μ*g/L	0.5–60 g/L
Repeatability	2.9%	3.8%
Reproducibility	5.2%	6.7%
Duration of a measurement	4-5 min	4-5 min

**Table 4:** Analyses of real samples.

Sample	Chronopotentiometry		AAS	
	As, *μ*g/L	Cd, g/L	As, *μ*g/L^a^	Cd, g/L^b^
A	<1	12.3 ± 1.1	<80	11.3 ± 0.7
B	57.5 ± 4.1	42.1 ± 1.9	<80	45.2 ± 2.5
C	67.2 ± 5.2	35.1 ± 2.1	<80	36.2 ± 1.9
D	154 ± 10.2	48.1 ± 3.7	130 ± 15	47.2 ± 3.0
E	165 ± 9.4	31.5 ± 2.0	180 ± 15	35.3 ± 1.8
F	193 ± 15.2	46.2 ± 2.9	210 ± 16	44.3 ± 2.2
G	440 ± 21.1	36.2 ± 2.1	670 ± 35	35.9 ± 1.7
H	740 ± 41.1	36.2 ± 2.1	1100 ± 65	35.9 ± 1.7

^a^Hydride generation AAS. ^b^Flame AAS. The samples were acquired from a cadmium production plant, Portovesme s. r. l., Portoscuso, Italy.
